# Neighborhood Socioeconomic Position and Social Support in a Clinical Population of Older Adults

**DOI:** 10.1093/geroni/igaa057.1276

**Published:** 2020-12-16

**Authors:** Kristen Berg, Adam Perzynski, Nikolas I Krieger, Douglas Einstadter, Jarrod Dalton

**Affiliations:** 1 Case Western Reserve University at MetroHealth, Cleveland, Ohio, United States; 2 Lerner Research Institute - Cleveland Clinic, Cleveland, Ohio, United States; 3 Cleveland Clinic Lerner College of Medicine at Case Western Reserve University, Cleveland, Ohio, United States

## Abstract

Models of successful aging underscore the critical role of external social resources in older adults’ health and well-being. Neighborhood socioeconomic position is known to influence health, but little is known about the linkages between neighborhood conditions, social relationships and health among older adults. We identified a cohort of 12,434 adults (aged 65+) who attended a Medicare Annual Wellness Visit from the NEOCARE Learning Health Registry. NEOCARE includes electronic health record (EHR) and neighborhood data from 1999-2017 on over 3 million unique Northeast Ohio individuals. The study population was 60% White, 32% Black or African American, 64% female, and 90% non-Hispanic. Over 60% were ages 65-74, 29% 75-84, and 10% 85 years or older (range from 65 to 101). We used ANOVA and chi square tests to examine variation in social support by quintile of the census tract area deprivation index. Compared to those in less disadvantaged neighborhoods, older adults in more disadvantaged neighborhoods were more likely to report needing help with care needs (Bonferroni-corrected x2=95.21, df=4, n=8,967, p<.001) but were less likely to report having help at home (x2=85.72, 4, n=12,354, p<.001). Similarly, adults in more disadvantaged neighborhoods reported less help available to them compared to those in more advantaged communities (F=39.31, df=4, n=12,099, p<.001). Furthermore, older adults living in more disadvantaged neighborhoods experienced significantly less functional independence (F=3.68, df=4 , n=8,571, p<.01). Our results suggest that the pathway from neighborhood socioeconomic conditions to successful aging includes perceived social needs and social support.

